# Host S100A6 inhibits ZIKV replication by degrading NS3 through lysosomal pathway

**DOI:** 10.3389/fcimb.2025.1602743

**Published:** 2025-10-01

**Authors:** Jiao Peng, WeiHao Zou, Lijuan Zhou, Runchun Liu, Hong-Juan Peng

**Affiliations:** ^1^ Department of Pathogen Biology, Guangdong Provincial Key Laboratory of Tropical Disease Research, School of Public Health, Southern Medical University, Guangzhou, Guangdong, China; ^2^ Guangxi Key Laboratory of AIDS Prevention and Treatment & Guangxi Universities Key Laboratory of Prevention and Control of Highly Prevalent Disease, School of Public Health, Guangxi Medical University, Nanning, Guangxi, China; ^3^ Guangxi Engineering Center for Organoids and Organ-on-chips of Highly Pathogenic Microbial Infections & Biosafety III Laboratory, Life Science Institute, Guangxi Medical University, Nanning, Guangxi, China

**Keywords:** Zika virus, S100 calcium binding protein A6, NS3 protein, virus replication, lysosomes

## Abstract

**Introduction:**

Zika virus (ZIKV) is a mosquito-borne arbovirus. Maternal infection may cause severe complications such as neonatal microcephaly and neurological defects. To date, there is no clinically approved vaccine or specific drug against ZIKV infection. The host calcium-binding protein, S100A6, is a member of S100 protein family, regulates various cellular processes, and has been recognized as a host-dependent factor for *Flavivirus* infection.

**Methods:**

S100A6 expression in host cells after ZIKV infection was detected by western blotting (WB). The effects of host S100A6 on ZIKV replication as indicated by the RNA and protein levels of nonstructural protein 3 (NS3) were detected by qRT-PCR, plaque assay, immunofluorescence assay (IFA), and WB respectively. The interaction and co-localization of S100A6 with NS3 were examined through co-immunoprecipitation (Co-IP) and IFA. Proteasome inhibitor and lysosomal acidification inhibitor were used to explore the degradation pathway of NS3 mediated by S100A6.

**Results:**

ZIKV infection induced a dose- and time-dependent increase in host S100A6 expression. Overexpression of S100A6 in HeLa cells did not affect ZIKV binding or entry into host cells but significantly inhibited viral replication. Conversely, S100A6 knockdown led to a significant increase in ZIKV replication. Moreover, S100A6 was found binding to ZIKV-NS3, leading to NS3 degradation without affecting genome copies. The use of lysosomal acidification inhibitor NH_4_Cl significantly reversed S100A6-mediated downregulation of NS3 protein levels, suggesting that S100A6 degrades NS3 via the lysosomal pathway.

**Conclusion:**

ZIKV infection upregulated host S100A6, which acted as an anti-infection factor by specifically targeting ZIKV-NS3 for degradation, thereby inhibiting viral replication. These findings provide insights into a potential mechanism of host resistance to ZIKV infection and enhance our understanding of the ZIKV-host interaction.

## 1 Introduction

In this decade, Zika virus (ZIKV) disease outbreaks in many countries and regions with large-scale ZIKV infections. In 2016, the World Health Organization (WHO) declared ZIKV infection a Public Health Emergency of International Concern (PHEIC) (Pielnaa et al., 2020). Infection in pregnant women may cause serious complications in fetus and newborns, such as neonatal microcephaly, congenital malformations, and neurological defects, while adults may suffer from serious complications including Guillain-Barre syndrome, meningitis, and retinopathy (Victora et al., 2016; Pierson and Diamond, 2018; Ferraris et al., 2019). With the increase of international travel and commercial exchanges around the world, the global spread of ZIKV has accelerated, posing a major threat to human health. On the other hand, no clinically approved specific vaccine or drug is available for treating ZIKV infection (Wang et al., 2022). Therefore, understanding the pathogenesis and host-virus interaction of ZIKV is urgent.

Zika virus (ZIKV) is an arbovirus transmitted by mosquitoes. Similar to other *flaviviruses*, ZIKV is a spherical enveloped virus with a diameter of about 50 nm (Sirohi and Kuhn, 2017). Its genome is a positive-sense single-stranded RNA of about 10.7 kb in length, encoding a single polyprotein. After processing and cleavage by both host and viral protease, it produces three structural proteins C, prM/M and E, and seven non-structural proteins NS1, NS2A, NS2B, NS3, NS4A, NS4B and NS5 (Sirohi and Kuhn, 2017). Viral structural proteins form the viral particles, and non-structural proteins contribute to viral genome replication and packaging, as well as to the regulation of host cells to support viral infection (White et al., 2016; Pierson and Diamond, 2018). NS3 is a multifunctional enzyme with a protease domain at the N-terminus and a helicase domain at the C-terminus (Hilgenfeld et al., 2018). The activity of NS3 protease requires NS2B as a co-factor to cleave ZIKV polyprotein, indirectly responsible for virus assembly and replication (Xing et al., 2020). The NS3 helicase domain is necessary for resolving the double-stranded RNA formed during viral gene synthesis (White et al., 2016).

The S100 protein family is a major subgroup of calcium-binding proteins, with 25 known members whose sequences and structures are highly conserved. They regulate a variety of cellular processes and functions (Donato et al., 2013; Chen et al., 2014; Gonzalez et al., 2020). S100A6 is expressed in different mammalian cells, tissues and several tumors, including neural, reproductive and placental tissues, which exhibits significant overlap with ZIKV target sites (Donato et al., 2017; Pierson and Diamond, 2018; Wang et al., 2023). S100A6 is involved in regulating numerous cellular functions, such as cell proliferation, apoptosis, cytoskeleton dynamics, and cell response to different stress factors (Bao et al., 2012; Donato et al., 2013, 2017; Lesniak et al., 2017; Song et al., 2020; Wang et al., 2021). Studies have found that S100A6 is upregulated in response to various stimuli, such as oxidative stress, arsenite exposure and viral infection (Shimizu et al., 2011; Wang et al., 2012, 2023). Intracellular viruses rely on host-related proteins for replication. S100A6 protein has been identified as a host-dependent factor for *Flavivirus* infection (Marceau et al., 2016). Studies have reported that S100A6 can inhibit hepatitis C virus (HCV) replication (Tani et al., 2013), and S100A9 can suppress highly pathogenic porcine reproductive and respiratory syndrome virus (HP-PRRSV) replication (Song et al., 2019). Given S100A6’s potential antiviral functions, its multifaceted roles in cellular regulation, and expression overlap with ZIKV target tissues, we prioritized it as a candidate host factor for mechanistic characterization of ZIKV infection.

In this study, we found that the expression level of host protein S100A6 was upregulated after ZIKV infection. As a host restriction factor of ZIKV, S100A6 inhibited viral replication and played an antiviral role through binding and degrading the viral NS3 protein.

## 2 Materials and methods

### 2.1 Cell culture and virus

HeLa, BHK-21, and COS7 cell lines were obtained from American Type Culture Collection (ATCC, Manassas, VA, USA) and maintained in Dulbecco’s Modified Eagles Medium (DMEM; Gibco/Invitrogen, Carlsbad, CA, USA) supplemented with 10% fetal bovine serum (FBS; Gibco/Invitrogen) and 1% Penicillin-Streptomycin (Gibco/Invitrogen) at 37°C and 5% CO_2_. *Aedes albopictus* C6/36 cells (ATCC) were maintained in Roswell Park Memorial Institute 1640 medium (RPMI 1640; Gibco/Invitrogen) supplemented with 10% FBS and 1% Penicillin-Streptomycin at 28°C. The Zika Virus strain Z16006 (GeneBank: no. KU955589.1) was kindly provided by the Institute of Microbiology, Guangdong Provincial Center for Disease Control and Prevention, and was propagated in C6/36 cells.

### 2.2 Plasmids

The ZIKV NS3 was cloned into pcDNA3.1(+) vector using a forward primer containing a KpnI restriction site and a reverse primer containing an XbaI restriction site, with a 3×FLAG tag fused at the C-terminal (The primers were listed in **Supplementary Table 1**). Complementary DNA (cDNA) was obtained by reverse transcription of RNA from the HeLa cells infected with ZIKV and used as the template for PCR amplification of ZIKV NS3 gene fragment. The amplified product was digested with restriction enzymes, ligated into the plasmid vector, and used to construct the recombinant expression plasmid. The resulting recombinant plasmids were confirmed by DNA sequencing (Tsingke Biotechnology, Beijing, China). The pcDNA3.1(+)-S100A6-HA and pcDNA3.1(+)-S100A6-TurboID-HA plasmids were previously constructed (Zhou et al., 2021) and stored in our laboratory at −20°C.

### 2.3 Antibodies and chemicals

The antibodies used in this study were anti-β-Actin antibody (Abcam, Cambridge, UK, Cat# ab179467), anti-ZIKV NS3 antibody (Genetex, CA, USA, Cat# GTX133309), anti-ZIKV E antibody (Genetex, CA, USA, Cat# GTX133314), anti-S100 alpha 6 antibody (Selleck, Houston, TX, USA, Cat# A5890), anti-HA antibody (Cell Signaling Technology, Boston, MA, USA, Cat# 3724), anti-FLAG M2 antibody (Sigma, St. Louis, MO, USA, Cat# F1804), goat anti-mouse IgG-HRP (ABclonal, Wuhan, China, Cat# AS003), goat anti-rabbit IgG-HRP (Abclonal, Wuhan, China, Cat# AS014), Alexa Fluor 594 goat anti-rabbit IgG (Invitrogen, Carlsbad, CA, USA, Cat# A32740), Alexa Fluor 488 goat anti-mouse IgG (Invitrogen, Carlsbad, CA, USA, Cat# A11001). The antibodies were used following the manufacturers’ guidance.

Proteasome inhibitor MG132 (APExBIO, Houston, TX, USA) was diluted in DMSO and added to the cell culture medium to a final concentration of 10 μM. Similarly, Lysosomal acidification inhibitor NH_4_Cl (aladdin, Shanghai, China) was diluted in sterile water and added to the cell culture medium to a final concentration of 30 mM.

### 2.4 Western blotting (WB)

Cells were collected and lysed on ice for 30 minutes using cell lysis buffer (Beyotime, Shanghai, China) containing 1mM phenylmethanesulfonyl fluoride (PMSF, Dingguo, China). After centrifugation at 14000×g, 4°C for 10 minutes, the supernatant was collected and the concentration was measured by bicinchoninic acid (BCA) method. For the S100A6-siRNA experiments, 100 μg of total protein was used, while for the other experiments, 50 μg of total protein was taken. The samples were then diluted in 6 × loading buffer, boiled for 10 minutes, then quickly placed in ice water. After a brief spin, the protein samples were loaded onto 10% or 15% SDS-PAGE gels for separation, and subsequently transferred to polyvinylidene fluoride (PVDF) membrane (Bio-Rad, Hercules, CA, USA). The membrane was blocked in 5% bovine serum albumin (BSA) dissolved in TBST (20 mM Tris-HCl, 150 mM NaCl, 0.1% Tween-20, pH 7.4) in a 37°C shaker for two hours, and then incubated with the primary antibody at 4°C overnight. After washing three times with TBST, the membrane was incubated with an HRP-labeled secondary antibody at 37°C for two hours. Finally, the protein bands were visualized using Clarity Western ECL Substrate (Bio-Rad) and photographed with a ChemiDoc Touch Imaging System (Bio-Rad).

### 2.5 RNA isolation and quantitative reverse transcription PCR (qRT-PCR)

The ZIKV copies, the knockdown and overexpression efficiency of S100A6, and the NS3 RNA copies were detected by qRT-PCR. Total RNA was isolated using TRIzol Reagent (Invitrogen) and the viral RNA in the supernatant of cell cultures was extracted according to the instructions of EasyPure Viral DNA/RNA Kit (TransGen, Beijing, China). Then the RNAs was reverse-transcribed to cDNAs using HiScript All-in-one RT SuperMix (Vazyme, Nanjing, China). The cDNA was subjected to quantitative PCR using Hieff qPCR SYBR Green Master Mix (Low Rox; Yeasen, Shanghai, China) on QuantStudio 6 real-time PCR system. The S100A6 transcript and NS3 RNA levels were normalized to the transcript level of glyceraldehyde-3-phosphate dehydrogenase (GAPDH), using comparative Ct method. Absolute quantification for ZIKV copies were implemented by comparison to a standard curve. The fragment corresponding to nucleotides (10,409 to 10,554) of ZIKV (Z16006) was used as an amplification target. Standard curves were prepared using 10-fold serial dilutions of known quantities of ZIKV fragment. All assays were performed in triplicate. The primers used to amplify the target genes are listed in **Supplementary Table 2**.

### 2.6 Viral binding and entry assay

HeLa cells were grown in a 12-well plate to about 70% confluence, and then transfected with pcDNA3.1(+)-S100A6-HA or control plasmid for 24 hours, using Lipofectamine 3000 Reagent (Invitrogen, USA) following the manufacturer’s instruction. For the virus binding assay, the cells were then infected with ZIKV at an MOI of 10 and incubated at 4°C for 1 hour. After the supernatant was discarded, the cells were washed with phosphate buffered saline (PBS) for three times. The cells were harvested and total RNA was extracted by TRIzol and the amount of the viral RNA was determined by qRT-PCR. For the entry assay, after one hour of ZIKV binding at 4°C, the cells were washed with PBS and incubated at 37°C for additional 1 hour. Subsequently, the supernatant was discarded and the cells were harvested following washing with PBS for three times. TRIzol was used to extract total RNA, and qRT-PCR was used to detect the effect of S100A6 on ZIKV entry. The efficiency of S100A6 overexpression was verified by WB.

### 2.7 plaque assay

BHK cells were seeded in 12-well plates until they reached approximately 70% confluency, and were transfected with either 3.0 ug siRNA-NC or siRNA-S100A6 per well for 24hours. Following transfection, cells were infected with ZIKV at an MOI of 0.1 and incubated for 5 or 10 days. The cells were then fixed in 10% paraformaldehyde for 5 min, stained with 0.1% crystal violet for 30min, followed by washing with PBS for 3 times. After that, the plaques on the plate bottom were photgraphed under a light microscope with 100× or 40× magnification.

### 2.8 Immunofluorescence assay

BHK-21 cells were grown on coverslips in a 12-well plate to about 70% confluence, and then transfected with 1 μg of plasmids using Lipofectamine 3000 Reagent (Invitrogen) following the manufacturer’s instruction for 24 hours. The cells were separated into two groups, and then infected with ZIKV for 24 hours or left uninfected for control. Afterward, cell cultures were aspirated, and cells were fixed and permeabilized with 100% precooled methanol for 8 minutes at −20°C. Then the methanol was aspirated and the cells were blocked with 10% BSA dissolved in PBS for one hour at room temperature.

After aspiration of the blocking buffer, the coverslips were incubated with the primary antibody at 4°C overnight, at the recommended concentration diluted in 10% BSA according to the manufacturer’s instruction. After washing three times with PBS, the coverslips were incubated with Alexa Fluor 594 goat anti-rabbit IgG (1:1500, Invitrogen) and/or Alexa Fluor488 goat anti-mouse IgG (1:1500, Invitrogen), diluted in 10% BSA for one hour at room temperature. Subsequently, the coverslips were washed with PBS for three times, rinsed with ddH_2_O and mounted with 4’,6-diamidino-2-phenylindole (DAPI) Fluoromount-G (Southern Biotech, USA). The samples were visualized and photographed using a fluorescence microscope (Nikon, Tokyo, Japan). Ten fields of view were randomly selected from the up, down, left, right and middle sections of each coverslip, and the total number of cells and the number of ZIKV positive cells were counted using Image J software. The virus infection rates of the two groups were calculated and compared.

### 2.9 Co-immunoprecipitation

HeLa cells were grown in T25 flasks to 70% confluence, and then, each flask was transfected with 3.0 µg pcDNA3.1(+)-S100A6-TurboID-HA or control plasmid for 24 hours using Lipofectamine 3000 Reagent (Invitrogen), following the manufacturer’s instruction. Afterward, the medium was replaced with DMEM containing 160 μM D-Biotin (Sigma-Aldrich, St. Louis, MO, USA) or no D-biotn supplemented, and the cells were infected with ZIKV at an MOI of 1, or left uninfected for another 24 hours. Then the cells were washed with PBS for three times, and lysed with cell lysis buffer (Beyotime) containing 1 mM phenylmethanesulfonyl fluoride (PMSF). Cell lysates were centrifuged at 14,000×g, 4°C for 10 minutes, and the supernatant was collected and incubated with Streptavidin magnetic beads (Thermo Fisher, Waltham, MA,USA) at 4°C overnight with gentle rotation. The beads were collected with a magnetic stand to remove the supernatant, and then, 500 μl precooled Washing Buffer (TBST, 0.1% Tween 20) was added and mixed gently with the beads. After washing three times, the magnetic beads were collected and resuspended in 50 μl PBS and 10 μl 6 × SDS loading buffer. The samples were boiled for 5 minutes and subjected to WB detection.

### 2.10 Statistical analysis

Statistical analysis was performed using GraphPad Prism 9 (GraphPad Software Inc., San Diego, CA, USA). A two-tailed unpaired Student’s t test was used to compare differences between each group, and one-way Analysis of Variance (ANOVA) with Tukey’s multiple comparisons test was used to compare differences among multiple groups. *P* < 0.05 was considered statistically significant. Data were represented as mean ± SEM, and all experiments were repeated at least three times.

## 3 Result

### 3.1 ZIKV infection upregulated S100A6 levels in host cells

**Figure 1:**
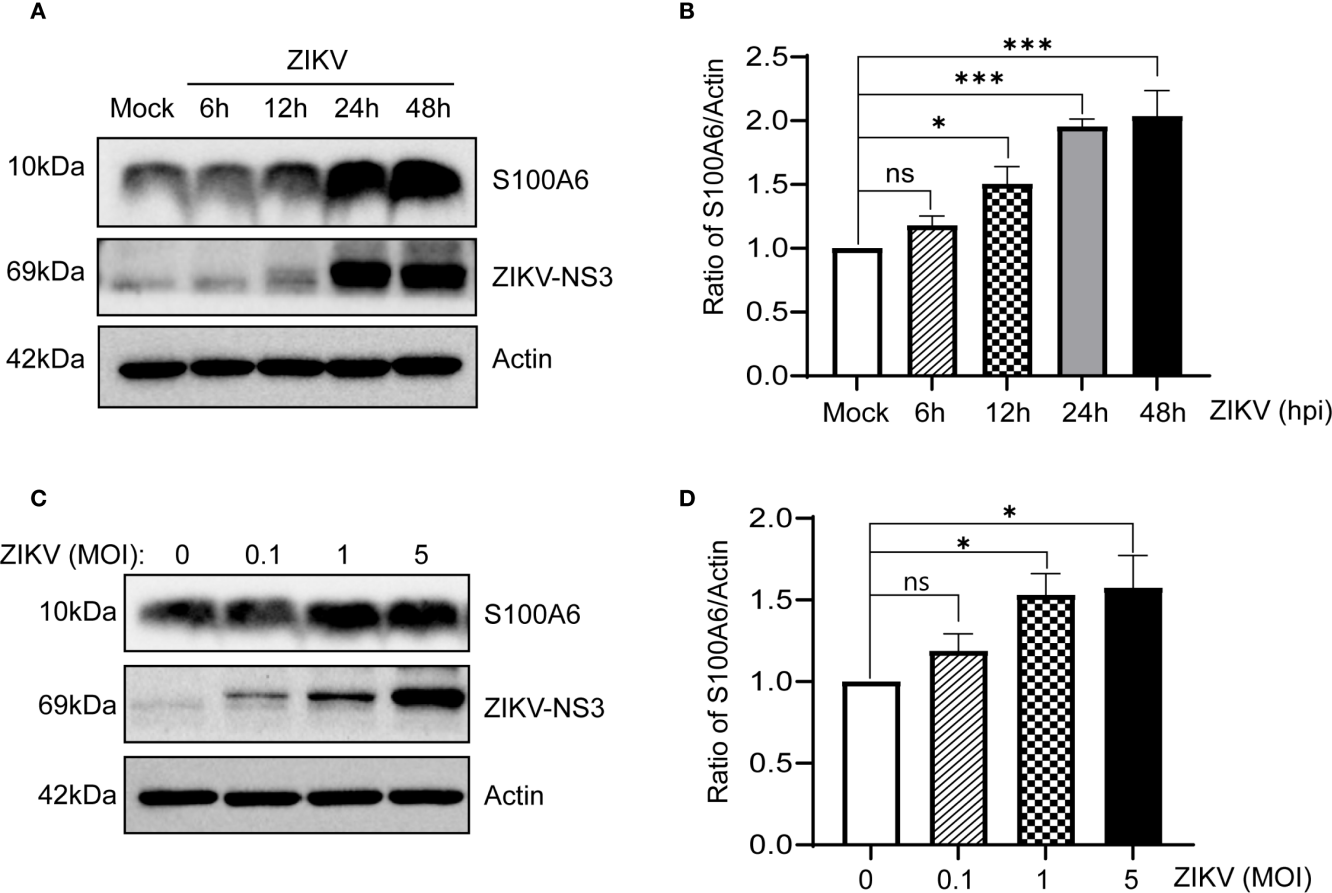
ZIKV infection increased S100A6 expression in HeLa cells. **(A, B)** HeLa cells were infected with ZIKV at an MOI of 1 for 6 h, 12 h, 24 h and 48 h The level of S100A6 protein was detected by Western blot (WB) and normalized to the level of β-actin using band density values by ImageJ software. Mock: uninfected control cells. **(C, D)** HeLa cells were infected with ZIKV at MOI of 0, 0.1, 1 and 5 for 24 h, respectively. Cell lysates were subjected to WB analysis for the indicated proteins, ImageJ software was used to quantify the density of S100A6 bands resulting from ZIKV infection at different MOIs. Data were analyzed by one-way ANOVA with Tukey’s *post hoc* test. ***P < 0.001; *P < 0.05; ns, not significant.

HeLa cells were infected with ZIKV at a multiplicity of infection (MOI) of 1 for 0 h, 6 h, 12 h, 24 h and 48 h, respectively. Cells were collected at the corresponding time points, and the expression of S100A6 was detected. The results showed that the S100A6 expression in ZIKV-infected cells was significantly increased. With the prolongation of infection time, the level of S100A6 increased in a time-dependent manner (**Figures 1A, B**). The faint NS3 signal observed in the Mock group represents background due to non-specific antibody binding. Subsequently, S100A6 levels were detected 24 hours post infection (hpi) with ZIKV at MOIs of 0, 0.1, 1 and 5 in HeLa cells. It was found that S100A6 expression was significantly upregulated with increasing MOIs in a dose-dependent manner (**Figures 1C, D**). These results demonstrated that ZIKV infection upregulated S100A6 protein levels in host cells, suggesting that S100A6 may be involved in the regulation of ZIKV infection.

### 3.2 S100A6 did not regulate ZIKV binding and entry into host cells

Given the increased expression of host S100A6 protein following ZIKV infection, we hypothesized that S100A6 might regulate the life cycle of ZIKV. The entire life cycle of ZIKV requires the interaction between viral and host proteins. During ZIKV infection, the attachment of viral envelope protein E to host receptors mediates its internalization into the host cell (Agrelli et al., 2019). Following the fusion of the virus membrane protein with host endosomal membrane, the viral genome is released into the cytoplasm to initiate viral genome replication and particle assembly.

**Figure 2:**
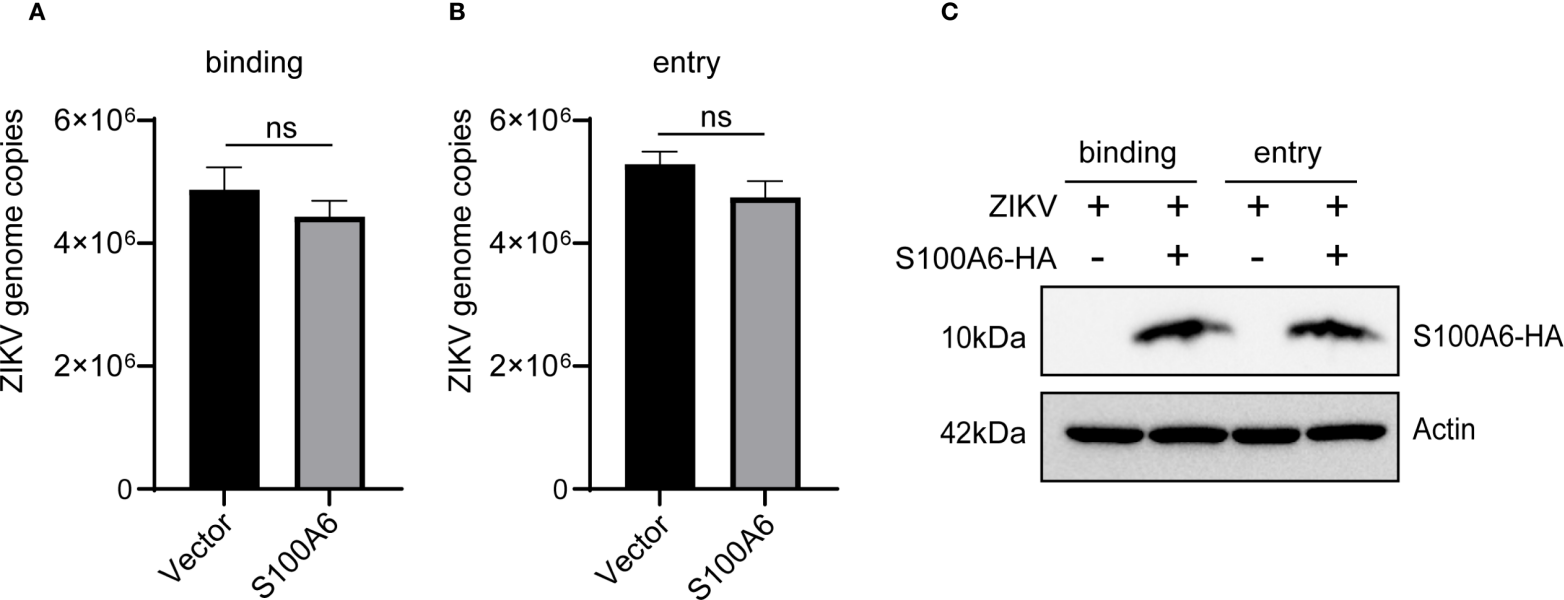
S100A6 overexpression did not affect ZIKV binding and entry into cells. HeLa cells were transfected with pcDNA3.1(+)-S100A6-HA or pcDNA3.1(+) for 24 h, and then infected with ZIKV at 4°C for 1 h (MOI = 10). **(A)** The cells were washed with PBS, and processed as follows: the cells were harvested and subjected to qRT-PCR for viral copy detection. **(B)** The cells were further incubated at 37°C for 1 h to internalize those binding viruses, and then harvested for viral copy detection with qRT-PCR. **(C)** WB was used to verify the overexpression of S100A6 protein. Student’s *t*-test was used for statistical test. ns, not significant.

To investigate whether S100A6 affects ZIKV’s binding and entry into host cells, HeLa cells were transfected with or without the pcDNA3.1(+)-S100A6-HA plasmid. To evaluate the impact of S100A6 on virus binding, cells were infected with ZIKV at 4°C for 1 h, washed three times with PBS and then harvested for qRT-PCR (**Figure 2A**). To determine whether S100A6 affected the virus entry, the HeLa cells were infected with ZIKV at 4°C for 1 h, then the cell culture medium was replaced with fresh medium, and the cells were incubated at 37°C for the internalization of the binding viruses (**Figure 2B**). The overexpression of the S100A6 protein was verified by WB (**Figure 2C**). There was no significant difference in viral copy number between the S100A6 overexpression group and the control group (**Figures 2A, B**), indicating that S100A6 did not affect virus binding and entry into cells.

### 3.3 Host S100A6 inhibited ZIKV replication

**Figure 3:**
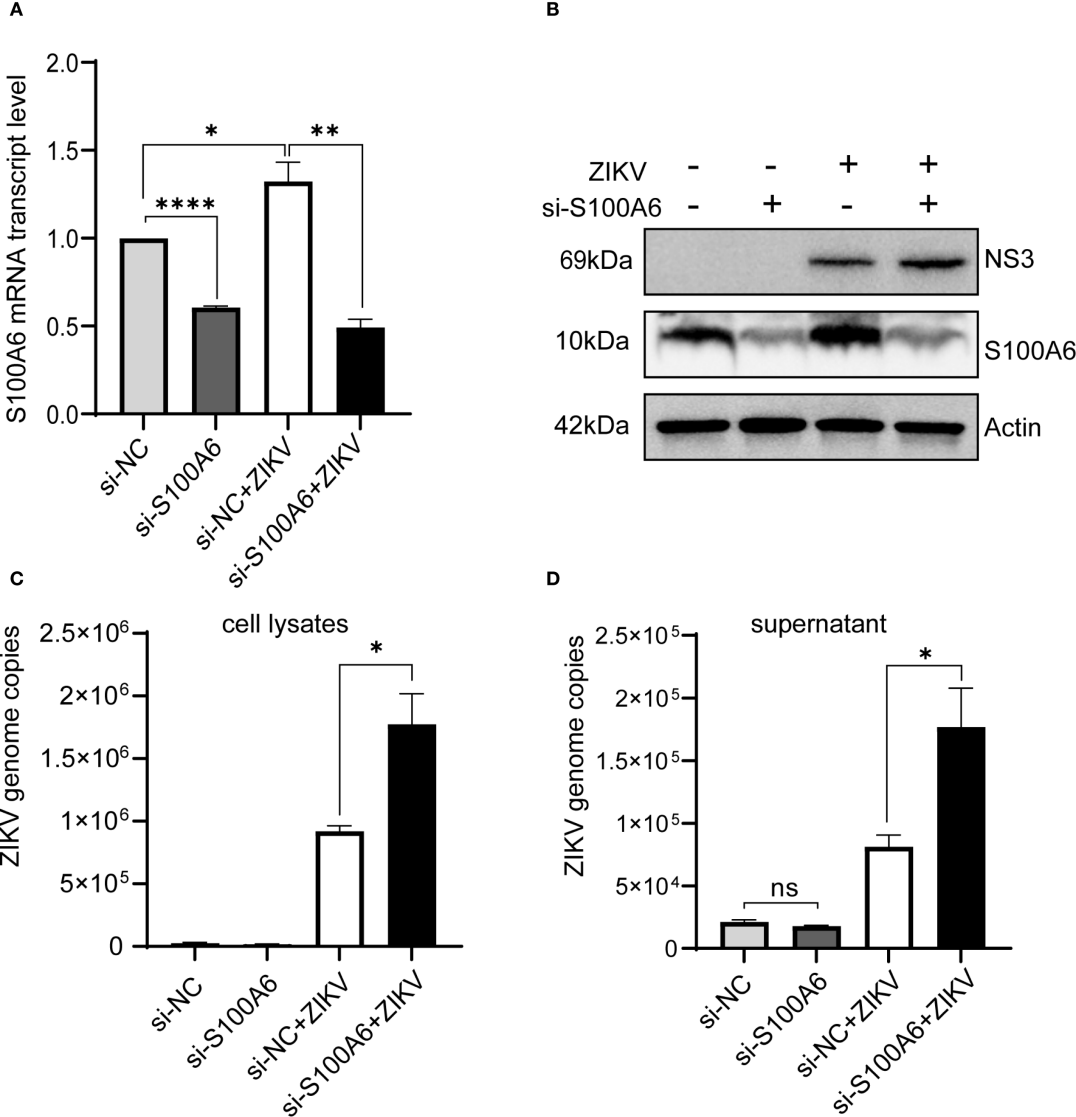
Knockdown of S100A6 increased ZIKV replication. HeLa cells were transfected with si-S100A6 or si-NC for 24 h, then challenged with ZIKV at an MOI of 1 and harvested at 24 hpi. **(A, B)** The knockdown efficiency of S100A6 was confirmed by qRT-PCR and WB. **(C, D)** The levels of cellular viral RNA and supernatant viral RNA were detected by qRT-PCR. Student’s *t*-test was used for statistical analysis. si-NC, negative control siRNA. ****P < 0.0001; **P < 0.01; *P < 0.05; ns, not significant.

Since S100A6 did not affect the binding and entry of ZIKV into host cells, we next explored whether it affected virus replication. RNAi was performed in HeLa cells to inhibit S100A6 expression, after which virus replication was detected. HeLa cells were transfected with si-S100A6 and si-NC for 24 hours, then infected with ZIKV for another 24 hours before being harvested for WB and qRT-PCR. The results showed that S100A6 expression level and transcription level were significantly reduced, confirming successful knockdown (**Figures 3A, B**). In cells transfected with si-NC, ZIKV infection increased S100A6 mRNA levels, which is consistent with the upregulation of S100A6 protein expression observed in **Figure 1**. After the knockdown of S100A6, the copy number of ZIKV in both the cell lysates and cell culture supernatant significantly increased (**Figures 3C, D**), suggesting that host S100A6 plays a role in inhibiting ZIKV replication.

**Figure 4:**
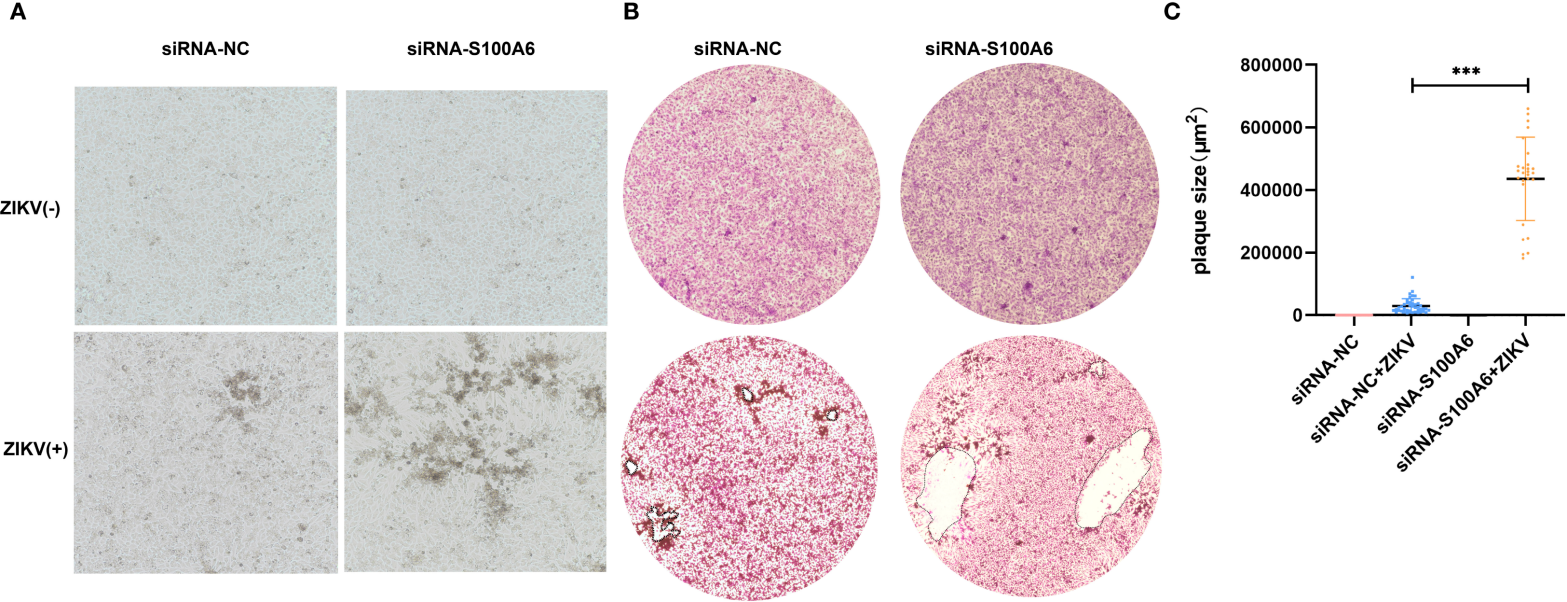
Identification of host S100A6 inhibiting ZIKV infection with plaque assay. BHK cells transfected with either siRNA-NC or siRNA-S100A6 for 24 h. ZIKV was added at an MOI of 0.1 for 5 days or 10 days. The cells were fixed with paraformaldehyde and stained with crystal violet. **(A)** Images of cytopathic effects observed at 5 dpi (100×). **(B)** Images of plaques formed at 10 dpi (40×). **(C)** Quantification of plaque areas using ImageJ software, and statistically compared across groups with a two-tailed unpaired Student’s t-test (***P < 0.001).

To further verify this, we performed plaque assays. At 5 days post infection (dpi), distinct patterns of cytopathic cell foci formation were observed across groups. Specifically, the si-S100A6 + ZIKV group exhibited the most pronounced foci, followed by the siRNA-NC + ZIKV group. No cytopathic foci were detected in the uninfected controls (siRNA-NC - ZIKV and si-S100A6 - ZIKV) (**Figure 4A**). At 10 dpi, the plaques formed in siRNA-S100A6 transfected cells were significantly larger than those in siRNA-NC transfected cells (**Figures 4B, C**).

**Figure 5:**
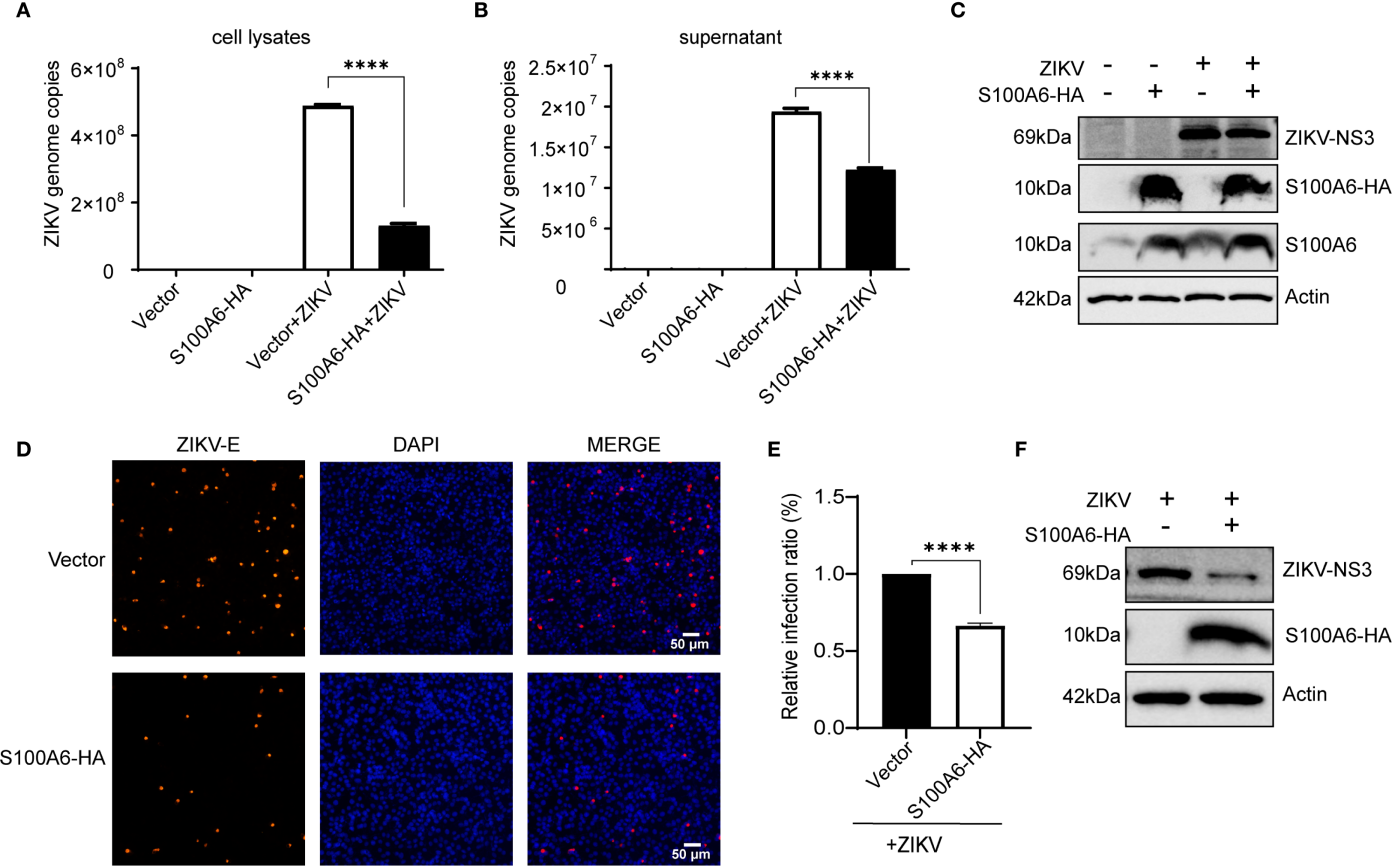
Overexpression of S100A6-HA reduced ZIKV replication. Cells were transfected with pcDNA3.1(+)-S100A6-HA or vector pcDNA3.1(+) for 24 h, then infected with ZIKV (MOI = 1) for 24 h. **(A, B)** The replication levels of ZIKV in cell lysates and cell supernatant were detected by qRT-PCR. **(C)** The expression levels of S100A6, S100A6-HA, NS3 and β-actin were analyzed by WB. **(D)** The virus was stained with a rabbit anti-ZIKV E antibody (red) and DAPI (blue) was used to stain nucleus (scale bar: 50 μm). **(E)** The number of cells and ZIKV-positive cells were counted by ImageJ software, and then the infection rates were calculated and compared. **(F)** The expression levels of S100A6-HA, NS3 and β-actin were detected by WB. Student’s *t*-test was used for statistical analysis. ****P < 0.0001.

Given that knockdown of S100A6 expression in host cells would lead to increased viral replication, to further explore the effect of S100A6 on viral replication, the pcDNA3.1-S100A6-HA plasmid was transfected into COS-7 cells, which were subsequently infected with ZIKV. S100A6 overexpression in cells was verified by WB with anti-HA antibody (**Figure 5C**), and ZIKV replication was detected by qRT-PCR. The copy numbers of ZIKV in both cell lysates and cell culture supernatants were significantly decreased after S100A6 overexpression (**Figures 5A, B**). We further confirmed the inhibitory effect of S100A6 on viral replication in BHK-21 cells by IFA. S100A6 overexpression was verified by WB with anti-HA antibody (**Figure 5F**). The nucleus was stained with DAPI (blue) and the virus was stained with a ZIKV E protein antibody (red). The total number of cells and ZIKV positive cells were counted by ImageJ software, and virus infection rates were calculated and compared. ZIKV infection rate in the S100A6 overexpression group was significantly lower than that in the control group (**Figures 5D, E**), consistent with the qRT-PCR results. These results further confirmed that the host protein S100A6 played a role in inhibiting ZIKV replication.

### 3.4 Host S100A6 promoted NS3 degradation

**Figure 6:**
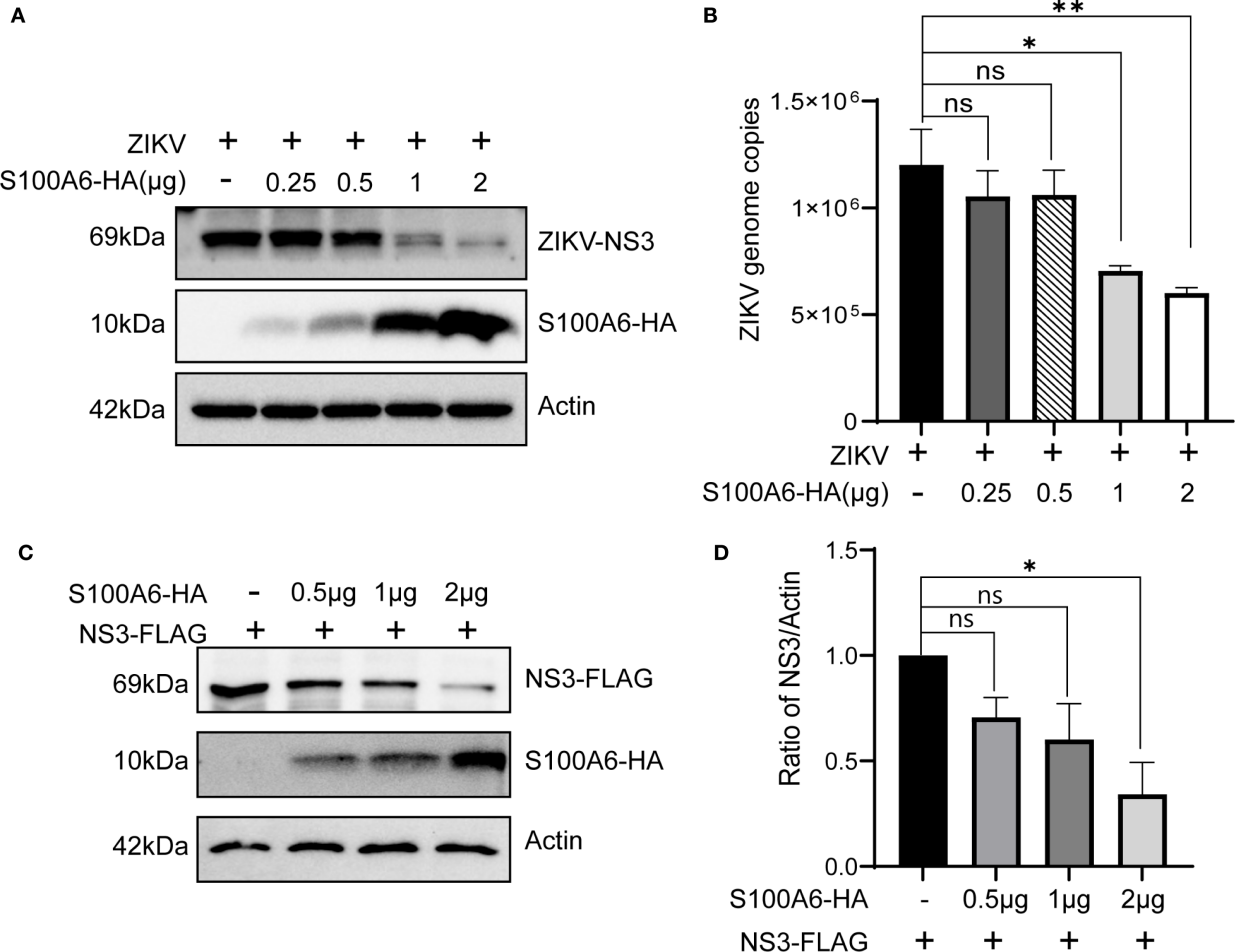
Increasing amounts of S100A6 decreased NS3 protein. **(A, B)** HeLa cells were transiently transfected with increasing amounts of S100A6-HA (0.25 μg, 0.5 μg, 1 μg and 2 μg). After 24 h of transfection, the cells were challenged with ZIKV at an MOI of 1 and harvested 24 hours post-infection. The expression levels of the indicated proteins were analyzed by WB, and the cellular Zika virus RNA copies was determined by qRT-PCR. **(C)** HeLa cells were co-transfected with pcDNA3.1-ZIKV NS3-FLAG and increasing amounts of pcDNA3.1-S100A6-HA (0.5 μg, 1 μg and 2 μg) for 48 hours, then cells were lysed and subject to WB for detection of the S100A6 and NS3 using anti-HA and anti-FLAG antibodies, respectively. **(D)** ImageJ software was used to quantify the density of the WB bands and to compare the ratio of FLAG to Actin in each group. For all overexpression transfections, vector pcDNA3.1(+) was used to ensure the balance of total DNA amount. One-way ANOVA was used for statistical analysis. *P < 0.05; **P < 0.01; ns, not significant.

NS3 is an essential protein for ZIKV replication. In our previous experiments, we found that S100A6 knockdown in HeLa cells resulted in a significantly higher NS3 protein level compared to the control group (**Figure 3B**). Conversely, overexpression of S100A6 led to a significant downregulation of the viral protein NS3 (**Figures 5C, F**). Furthermore, HeLa cells were transfected with a gradient of S100A6-HA plasmid and then infected with ZIKV for 24 hours. As S100A6 protein expression increased, ZIKV copies decreased significantly in a dose-dependent manner (**Figure 5A**). Meanwhile, we also found that the level of ZIKV NS3 protein and ZIKV genome copies decreased significantly with the increase of S100A6 (**Figures 6A, B**).

Therefore, we hypothesized that S100A6 might inhibit viral replication through its effect on the NS3 protein. We further constructed the pcDNA3.1(+)-NS3-FLAG plasmid, and co-transfected it with pcDNA3.1(+)-S100A6-HA into HeLa cells. It was found that the exogenous NS3 protein level decreased with an increased amount of S100A6 (**Figures 6C, D**), indicating that ZIKV NS3 levels were negatively correlated with S100A6 protein amounts and that S100A6 promotes the degradation of ZIKV NS3.

### 3.5 S100A6 degraded NS3 protein through the lysosomal pathway

**Figure 7:**
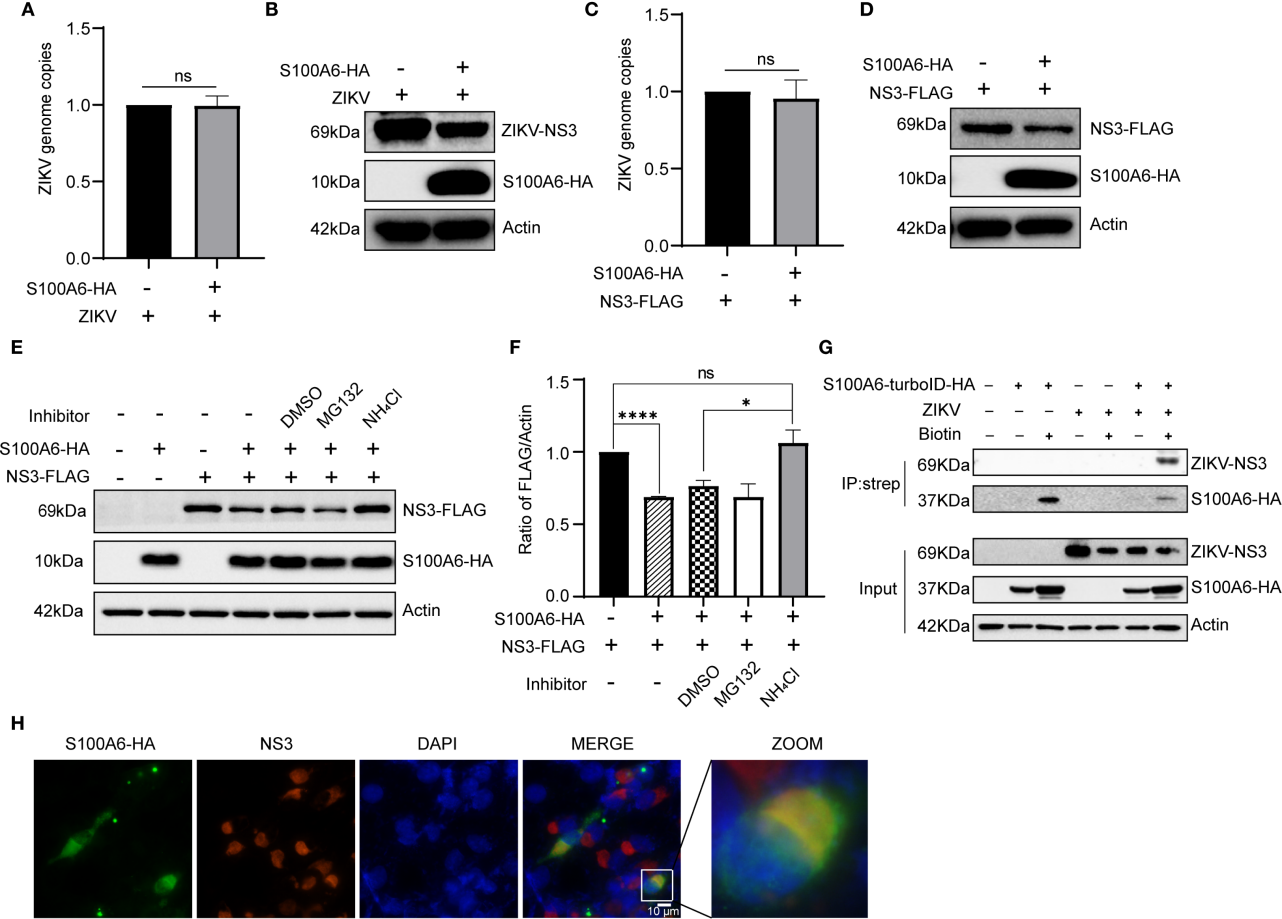
S100A6 promoted the degradation of NS3 protein through the lysosomal pathway. **(A, B)** HeLa cells were transfected with pcDNA3.1(+)-S100A6-HA or pcDNA3.1(+) for 24 h, then infected with ZIKV (MOI = 1) for 24 h. The genome copy was detected by qRT-PCR, and the protein level was detected by WB. **(C, D)** HeLa cells were transfected with pcDNA3.1(+)-NS3-FLAG alone or co-transfected with pcDNA3.1(+)-S100A6-HA for 48 h, then the genome copy was detected by qRT-PCR, and the protein levels were detected by WB. **(E, F)** HeLa cells were transfected with pcDNA3.1(+)-NS3-FLAG alone, or co-transfected with pcDNA3.1(+)-S100A6-HA, and treated with DMSO, MG132 (10 μM) or NH_4_Cl (30 mM) for 12 h, respectively, or not treated. The level of NS3 was detected by WB and normalized to β-actin by ImageJ software. **(G)** Lysates of HeLa cells overexpressing S100A6-turboID-HA and infected with ZIKV were immunoprecipitated with streptavidin magnetic beads. The immunocomplexes were analyzed with the indicated antibodies by WB. **(H)** co-localization of S100A6 and ZIKV NS3 in BHK-21 cells were visualized by IFA. S100A6-HA was detected with an anti-HA antibody and visualized with the Alexa Fluor 488 (green), and NS3 was detected with an anti-NS3 antibody and visualized with the Alexa Fluor 594 (red). The co-localization is shown in yellow (scale bar: 10 μm). ****P < 0.0001; *P < 0.05; ns, not significant.

S100A6 downregulated the NS3 protein level, but it was not clear whether this was regulated at RNA or protein level. We overexpressed S100A6 in HeLa cells, then infected them with ZIKV(**Figures 7A, B**), or simultaneously overexpressed both S100A6 and NS3(**Figures 7C, D**). We found that the protein level of NS3 was significantly decreased compared to the control group without S100A6 expression in both conditions (**Figures 7B, D**). However, the genome copy (represented by NS3-RNA copy), as detected by qRT-PCR, was not significantly different from the control group (**Figures 7A, C**), indicating that the downregulation of NS3 protein level by S100A6 occurred at the post-translational level rather than the RNA level.

In eukaryotic cells, proteins are mainly degraded through the ubiquitin-proteasome pathway and lysosome pathway. To explore which pathway S100A6 utilized to degrade NS3, we co-expressed S100A6-HA and NS3-FLAG in HeLa cells. After 36 hours of transfection, the cells were treated with either the proteasome inhibitor MG132 or the lysosomal acidification inhibitor NH_4_Cl for 12 h, and the level of NS3 protein was detected by WB. NS3 degradation was still observed in the MG132-treated group, whereas NH_4_Cl restored the decrease in NS3 level caused by S100A6 (**Figures 7E, F**). These results indicated that overexpression of S100A6 promoted NS3 protein degradation through the lysosomal pathway.

### 3.6 Host S100A6 interacted with NS3

Host S100A6 degraded ZIKV NS3 through the lysosomal pathway. We further investigated whether this degradation process was facilitated by the interaction between S100A6 and NS3. HeLa cells were infected with ZIKV following S100A6 overexpression, and the cells were lysed for Co-IP. WB results showed that S100A6 interacted with ZIKV NS3 (**Figure 7G**). Next, we used IFA to detect the co-localization of S100A6 and NS3 in cells. BHK-21 cells were transfected with S100A6-HA, infected with ZIKV, and stained with a fluorescent HA antibody (green) and NS3 antibody (red). Co-localization was indicated by yellow fluorescence. We observed co-localization of S100A6 and NS3 in the cytoplasm (**Figure 7H**). Therefore, we concluded that S100A6 co-localized and interacted with ZIKV NS3, inducing NS3 degradation through the lysosomal pathway.

## 4 Discussion

ZIKV can effectively infect and replicate in various cell types. However, because its small RNA genome encodes only 10 proteins, ZIKV is highly dependent on host factors for replication, RNA synthesis and assembly of viral particles (Nagy and Pogany, 2011). ZIKV can promote or inhibit the expression of some host proteins involved in the process of its binding, entry and replication (Zhou et al., 2019; Yang et al., 2020). It co-localizes with host Hsp70 and promotes viral RNA production and viral particle release (Pujhari et al., 2019). BiP interacts with viral envelope protein E and cellular alkaline phosphatase, thus promoting its infection (Chen et al., 2020). Host protein S100A6 is involved in the regulation of multiple cellular processes and is upregulated in response to various stimuli (Tamai et al., 2011; Donato et al., 2017; Biji et al., 2021). In porcine alveolar macrophages infected by HP-PRRSV, S100A6 is significantly upregulated (Zhou et al., 2015). *Haemophilus parasuis* infection significantly increases the transcription levels of S100A4 and S100A6 genes in the lungs, spleen and lymph nodes of pigs (Wang et al., 2012). Similarly, we found that ZIKV infection significantly promoted the expression of host protein S100A6 in a dose- and time-dependent manner. The S100 protein family can interact with various pathogenic proteins, influencing their infection and replication processes. For example, S100A6 inhibits hepatitis C virus replication by interacting directly with FKBP8/FKBP38 and regulating the NS5A-FKBP8/FKBP38 interaction (Tani et al., 2013).

In *Toxoplasma gondii* infection, S100A6 interacts with surface antigen 1 (SAG1), promoting adhesion, invasion, and infection (Zhou et al., 2021). S100A9 significantly inhibits the replication of HP-PRRSV by interacting with the viral N protein (Song et al., 2019). In our study, we observed that ZIKV infection upregulated S100A6 expression, and S100A6 knockdown in HeLa cells led to increased viral replication. Conversely, overexpression of S100A6 significantly inhibited viral replication but did not affect the binding and entry of the virus.

ZIKV structural proteins and genomic RNA form viral particles, and non-structural (NS) proteins are necessary for viral RNA replication, particle assembly, and inhibition of the host’s antiviral innate immune response (Pierson and Diamond, 2018). NS proteins play important roles in the life cycle of the virus and the pathogenesis of ZIKV. Among the non-structural proteins, NS3 is a multifunctional protein composed of an N-terminal protease domain and a C-terminal helicase domain, which is involved in the unwinding of double-stranded RNA and the cleavage of ZIKV polyprotein during viral replication. Due to the multiple roles of NS3 in the viral cycle, it has been one of the main targets for drug screening in recent years (Li et al., 2016; Tay and Vasudevan, 2018). ZIKV infection can induce the expression of C19orf66 in cells, which interacts and co-localizes with ZIKV NS3, thereby inducing NS3 degradation through a lysosome-dependent pathway and inhibiting viral infection (Wu et al., 2020). PARP12 interacts with ZIKV NS1 and NS3, mediating their degradation through the proteasome pathway, and inhibiting viral replication (Li et al., 2018). In our study, we found that the host protein S100A6 led to the degradation of ZIKV NS3, suggesting that its downregulation may be related to ZIKV pathogenicity. In addition, S100A6 interacted and co-localized with ZIKV NS3, forming a complex in the infected cells.

Proteins are mainly degraded by the ubiquitin-proteasome pathway or lysosomal pathway. The lysosome is a membrane-bound organelle in eukaryotic cells containing various hydrolases, breaks down endogenous and exogenous biomolecules, including proteins, nucleic acids, carbohydrates, lipids and cell debris (Saftig and Klumperman, 2009). It is essential for innate immune recognition, antigen presentation and pathogen elimination (Spence et al., 2019). ZIKV NS3 can mediate the cleavage of the endoplasmic reticulum autophagy receptor FAM134B to inhibit autophagy to achieve effective viral replication and viral particle assembly (Lennemann and Coyne, 2017). In our study, the lysosomal acidification inhibitor NH_4_Cl treatment significantly inhibited S100A6-mediated NS3 degradation, indicating that S100A6 degraded ZIKV NS3 in a lysosomal-dependent manner, thus interfering with the life cycle of the virus and inhibiting viral replication. In human histiocytic lymphoma cells (U937), PKC-δ translocates to lysosomes, mediating the phosphorylation and activation of lysosomal acidic sphingomyelinase, and activates the lysosomal degradation pathway (Parent et al., 2011). It is also reported that S100 protein and PKC are co-localized (Lefranc et al., 2005). And NH4Cl is a weak base known to inhibit lysosomal hydrolases by reducing the acidification of the lysosomal compartment. Future studies should thus explore the interaction mechanisms of lysosome-related molecules with S100A6 in the degradation of ZIKV NS3 protein. We hypothesized that S100A6 may mediate the degradation of ZIKV NS3 by forming a complex with PKC or other enzymes, or through interacting with lysosome-associated molecules.

Due to the multiple cellular functions of S100A6, besides binding and degrading NS3, S100A6 may also indirectly affect ZIKV replication by regulating cytoskeleton proteins such as vimentin (Zhang et al., 2022), interacting with calcium cycle protein binding protein/Siah-1 interacting protein (CacyBP/SIP) (Donato et al., 2017), and regulating tumor suppressor factor p53 and cyclin-dependent kinase (CDK) (Bao et al., 2012), thereby regulating cell cycle, proliferation, apoptosis, migration and other cellular processes. Host factors are potential therapeutic targets for various viral infections (Brass et al., 2009; Zhang et al., 2016). Although the interaction between ZIKV and its host cell remains to be further explored, we found that ZIKV infection increased the expression of S100A6 in host cells, and S100A6 could significantly inhibit virus replication. S100A6 interacted with and co-localized with ZIKV NS3, and induced NS3 degradation through the lysosomal pathway.

In summary, S100A6 acts as a host restriction factor, exerting an antiviral effect against ZIKV by degrading NS3 through the lysosomal pathway. These findings advance our understanding of the molecular mechanisms underlying ZIKV pathogenesis and may guide the development of novel antiviral therapeutic targets.

## 5 Conclusion

ZIKV infection promoted the expression of host S100A6 protein, which in turn regulated ZIKV NS3 degradation through the lysosomal pathway, thereby inhibiting viral replication. Our study highlights the role of S100A6 in ZIKV infection, revealing a novel mechanism for host resistance to the virus. These findings enhance our understanding of the interaction between ZIKV and its host and provide a theoretical basis for further investigation into the virus’s pathogenic mechanisms.

## Data Availability

The original contributions presented in the study are included in the article/**Supplementary Material**. Further inquiries can be directed to the corresponding author.
