# Supplementary material for: Host S100A6 inhibits ZIKV replication by degrading NS3 through lysosomal pathway
Source: Front Cell Infect Microbiol. 2025 Oct 1;15:1602743. doi: 10.3389/fcimb.2025.1602743 (PMC12521416; doi:10.3389/fcimb.2025.1602743)
Supplement: Supplementary file 1 [file Table1.docx]

***Supplementary Material***

**1 Supplementary Figures and Tables**

**Supplementary Table 1.** Primers used for construction of full length of NS3 with 3×FLAG-tag.

| **Target** | **Primers (5’-3’)**  **F: Forward R: Reverse** |
| --- | --- |
| NS3 full length-F | CGGGGTACCAGTGGAGCTCTATGGGATG |
| NS3 full length-R1 | ATCATGATCCTTGTAGTCTCCGTCGTGGTCCTTATAGTCTCTTTTCCCAGCGGCAAAC |
| NS3 full length- R2 | TGCTCTAGACTACTTATCGTCATCGTCTTTGTAATCAATATCATGATCCTTGTAGTCTCCGTC |

(3×FLAG-tag sequence: GACTATAAGGACCACGACGGAGACTACAAGGATCATGATATTGATTACAAAGACGATGACGATAAG)

**Supplementary Table 2.** Primers used for qRT-PCR.

| **Target** | **Primers (5’-3’)**  **F: Forward R: Reverse** |
| --- | --- |
| ZIKV-F | CTATAGTCAGGCCGAGAACGC |
| ZIKV-R | CCCAGATTAAAGGGTGGGGA |
| NS3-F | GCCTAAAGCCGGTCATACTT |
| NS3-R | ACCCACCTCCATACAGATACT |
| GAPDH-F | GTCAACGGATTTGGTCGTATTG |
| GAPDH-R | TGTAGTTGAGGTCAATGAAGGG |
| S100A6-F | AAGGCTGATGGAAGACTTGG |
| S100A6-R | CCTTGAGGGCTTCATTGTAGAT |
